# Gut Microbiota and Its Metabolite Taurine-*β*-Muricholic Acid Contribute to Antimony- and/or Copper-Induced Liver Inflammation

**DOI:** 10.3390/ijms26073332

**Published:** 2025-04-03

**Authors:** Dandan Wu, Qiwen Lin, Senao Hou, Xiaorui Cui, Na Shou, Xuefeng Yuan, Wenqian Xu, Keyi Fu, Qi Wang, Zunji Shi

**Affiliations:** State Key Laboratory of Herbage Improvement and Grassland Agro-Ecosystems, Center for Grassland Microbiome, College of Pastoral Agriculture Science and Technology, Lanzhou University, Lanzhou 730000, China; wudd2019@lzu.edu.cn (D.W.); linqiwen1005@163.com (Q.L.); housa20@lzu.edu.cn (S.H.); cuixr2023@lzu.edu.cn (X.C.); shoun19@lzu.edu.cn (N.S.); 220220901580@lzu.edu.cn (X.Y.); xuwq2023@lzu.edu.cn (W.X.); fuky2018@lzu.edu.cn (K.F.); wangqiyjs@163.com (Q.W.)

**Keywords:** antimony, copper, metabolomics, bile acids, liver inflammation

## Abstract

Antimony and copper can contaminate vegetables and enter the human body through the digestive tract, inducing severe and extensive biotoxicity. However, the role of bile acids (BAs) in the pathogenesis of liver inflammation by antimony or copper has not been elucidated. Our results indicated that antimony and/or copper induced liver inflammation, causing the disruption of gut microbiota, with the down-regulation of probiotics and up-regulation of harmful bacteria closely correlated to liver inflammation. Targeted metabolomics of BAs showed that antimony and/or copper significantly up-regulated the levels of taurine-*β*-muricholic acid (T-*β*-MCA) in serum and liver, which was due to the reduction of Lactobacillus spp. A farnesoid X receptor (FXR) antagonist, T-*β*-MCA inhibited the FXR-SHP pathway in liver and FXR-FGF15 pathway in ileum, thereby promoting the transcription of cholesterol 7-alpha hydroxylase (CYP7A1) and increasing total bile acid concentrations, ultimately leading to liver inflammation. These findings provide new insights into the underlying mechanisms of antimony- and/or copper-induced liver inflammation.

## 1. Introduction

Antimony and copper are globally important environmental pollutants with severe and widespread biotoxicity. Copper and antimony are widely recognized as environmental contaminants [1]. Copper and antimony pollution in plants and soil is mostly caused by mining, industrial emissions, fossil fuel combustion, metal aerosols, untreated sewage, and sludge irrigation [2]. Trace metals can move from soil to crops and may constitute a health risk to consumers [3]. With the development of urban agriculture, an increasing number of people are exposed to antimony and copper. The soil concentration of copper and its availability to crops can affect crop health, which, in turn, affect animal health [4]. Antimony can accumulate in the edible sections of plants after being transmitted from the soil or atmosphere [5]. The interference of heavy metals including antimony or copper to the diversity and composition of the intestinal microbiota may affect the metabolic and physiological functions, thereby causing damage to the host and accelerating the course of multiple diseases [6]. According to previous research, antimony toxicity can cause adverse effects on the respiratory system, liver, kidneys, and cardiovascular system [7]. Acute copper poisoning may lead to gastrointestinal disorder, hemolytic anemia, and organ damage, while chronic exposure can affect the liver, kidneys, and nervous system [8]. Researchers have found that exposure to antimony can induce significant hepatotoxic damage at levels of 10 mg/kg [9], and copper can induce the histopathological inflammation and apoptosis of liver in mice at levels of 100 mg/kg [10].

Bile is mainly composed of bile acids (BAs), originating from cholesterol in the liver. These BAs are categorized into primary and secondary types. The liver synthesizes primary BAs, notably cholic acid (CA) and chenodeoxycholic acid (CDCA) [11]. In contrast, secondary BAs are derived from primary BAs through the action of gut microbiota, including deoxycholic acid (DCA), ω-muricholicacid (*ω*-MCA), and lithocholic acid (LCA) [12]. The liver synthesizes primary BAs from cholesterol via cholesterol 7-*α*-hydroxylase (CYP7A1), which are subsequently conjugated with glycine or taurine to improve solubility [13]. BAs are subsequently excreted into the bile and biotransfomed by gut microbiota, resulting in the production of secondary BAs. Notably, the increased contents of BAs in serum were closely correlated with liver inflammation [14].

The farnesoid X receptor (FXR) known as the bile acid receptor (BAR) can regulate BA levels and inflammatory responses in enterohepatic circulation [15]. Specifically, FXR plays a crucial role in modulating glucose metabolism, exhibiting a molecular link between BAs and glucose metabolism [16]. As a signaling pathway, BAs can produce metabolic effects through its interaction with the nuclear receptor FXR [17]. The hydrophobic CDCA is the most potent FXR ligand, while hydrophilic BAs, including muricholic acid (MCA) and ursodeoxycholic acid (UDCA), cannot activate FXR [18]. DCA and tauro-beta-muricholic acid (T-*β*-MCA) are the main antagonists of FXR [19]. Notably, the FXR-SHP signaling pathway is composed of nuclear receptor FXR and its linear target protein a “small heterodimer partner” (SHP), regulating BA levels and BA homeostasis [20].

The disturbance of the gut microbiota disrupts secondary BAs. The dysregulation of the gut microbiota can cause intestinal inflammation and other intestinal immunological alterations, eventually leading to liver inflammation [21]. It has been well-documented that the gut microbiota plays a pivotal role in determining the composition of the BAs pool in the gut. BAs are known to engage in the gut–liver axis by modulating the FXR, which influences both the BAs and the microbiota [22]. The composition of gut microbiota can impact bile acid profiles and ameliorate liver damage in mice with a bile acid composition similar to humans [23]. The interplay between the gut and the liver, known as the gut–liver axis, is crucial for managing gastrointestinal health and diseases. Nutrients, metabolites, and BAs can influence the metabolism and immune responses of both the gut and the liver, as well as the structure and function of gut microbiota [24].

Antimony and copper are common environmental and industrial pollutants encountered by humans, which may pose a threat to human health and cause hepatic toxicity. Researchers have found that exposure to antimony can induce significant hepatotoxic damage at levels of 10 mg/kg [9], and copper can induce histopathological inflammation and apoptosis of liver in mice at levels of 100 mg/kg [10]. However, it is unclear whether antimony and copper cause liver damage through the enterohepatic circulation. Thus, we hypothesize that antimony and/or copper might have comparable and distinct impacts on liver inflammation through the enterohepatic circulation. The effects of antimony, copper, antimony + copper (SC) on gut microbiota and its derivative BAs, as well as the FXR-SHP signaling pathway, were evaluated by high-throughput sequencing, targeted metabolomic analysis, and quantitative real-time PCR analysis (qPCR) in mice.

## 2. Results

### 2.1. Identification of Liver Inflammation Phenotype Induced by Antimony and/or Copper

**Figure 1:**
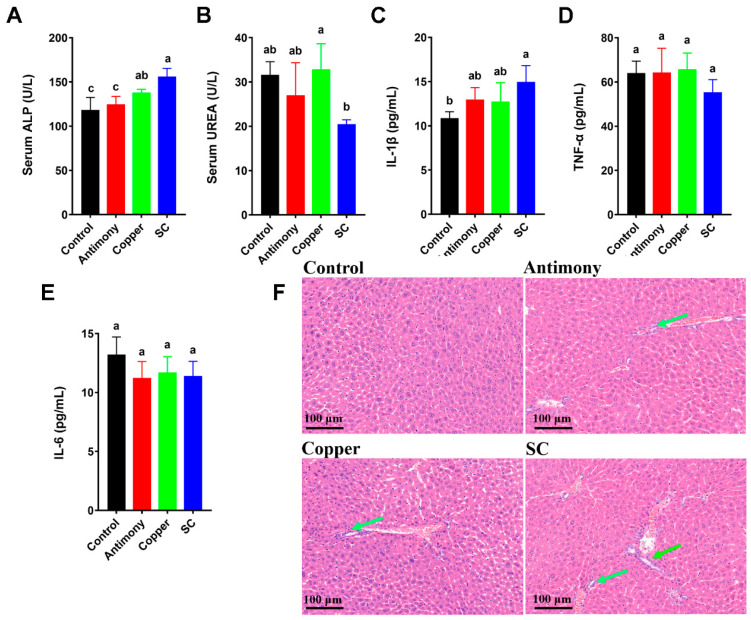
Identification of inflammatory phenotypes in liver caused by antimony and/or copper. (**A**,**B**) Serum biochemical markers including ALP and UREA. (**C**–**E**) Hepatic IL-1β, TNF-α, and IL-6 levels by ELISA. (**F**) Microscopic images of H&E-stained liver tissues (×400) from the control, the antimony, the copper, and the SC groups. Green arrows highlight areas of hepatocyte nuclear condensation. Results are expressed as mean ± standard deviation with *n* = 7 per group. Scale bars in panel C represent 100 μm. In bar graphs (**A**–**E**), different letters denote statistically significant differences as determined by one-way ANOVA (*p* < 0.05).

After six weeks of treatment with antimony and/or copper, there were no significant changes in liver weight, liver/body weight ratio, body weight, and food intake (Figure S1). The degree of liver inflammation was assessed by analyzing serum biochemical indices, showing that the serum ALP level in the copper group significantly increased by 1.3-fold, while the serum UREA level in the SC group significantly decreased by 1.6-fold (Figure 1A,B). Other serum biochemical indices (ALT, AST, HDL-C, and TG) had no significant changes between pairwise comparisons, except that the serum GLU level was remarkably elevated in the SC group, and the serum CREA level was significantly reduced in the SC group compared to the control group (Figure S2). In addition, compared with the control group and antimony group, the serum D-BIL level in the copper group was significantly increased, and compared with the antimony group, the serum T-BIL level in the copper group was significantly increased (Figure S2). The H&E-stained results indicated that the inflammatory cell infiltration and nuclear condensation of hepatocytes were found in all three treatment groups, and the phenotype was more pronounced in the SC group (Figure 1F). In addition, the level of pro-inflammatory cytokine IL-1*β* in the liver of the SC group was significantly higher by 1.4-fold than that of the control group, but there were no significant differences in TNF*-α* and IL-6 (Figure 1C–E). The above results showed that antimony and/or copper treatments caused liver inflammation.

### 2.2. Disrupted BA Pools in Serum and Liver

**Figure 2:**
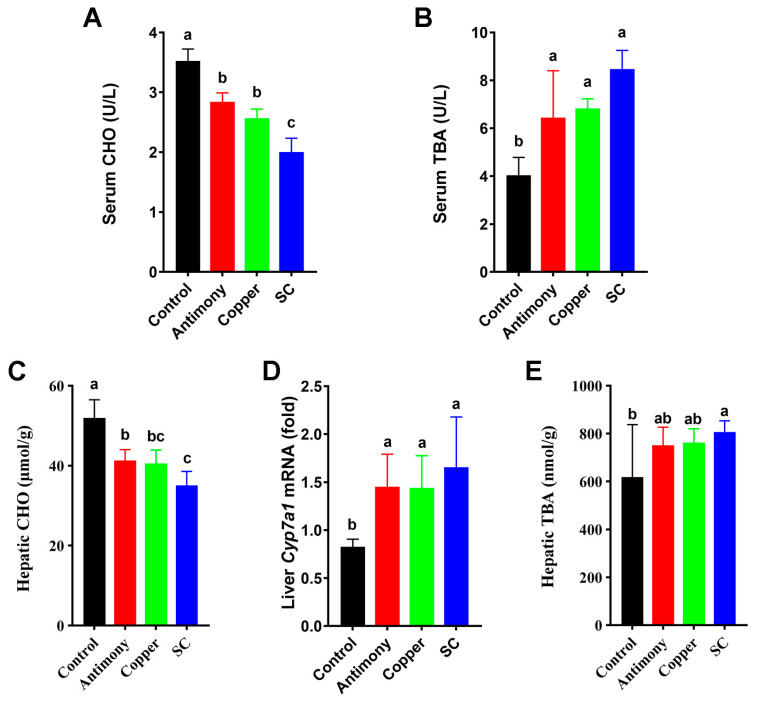
Quantitative analysis of BAs biosynthesis from cholesterol. (**A**) ELISA analysis of serum CHO. (**B**) Determination of serum biochemical indices including serum TBA. (**C**,**E**) ELISA analysis of hepatic CHO and TBA. (**D**) Hepatic *Cyp7a1* mRNA expression level. Presented data are the average ± standard deviation for each group with *n* = 7. Bar graphs (**A**–**E**) marked with distinct letters signify statistically significant differences according to one-way ANOVA at *p* < 0.05.

Serum CHO and hepatic CHO were significantly reduced in the antimony group and the copper group by 1.3-fold and in the SC group by 1.7-fold (Figure 2A,C), serum TBA was markedly elevated in the antimony group and the copper group by 1.6-fold and in the SC group by 2-fold (Figure 2B), and hepatic TBA was significantly elevated in the SC group compared to the control group (Figure 2E). The expression of *Cyp7a1* mRNA in the liver of mice was significantly elevated in all three treatment groups by 2-fold (Figure 2D); notably, *Cyp7a1* is the first restriction endonuclease catalyzing the production of BAs from cholesterol.

**Figure 3:**
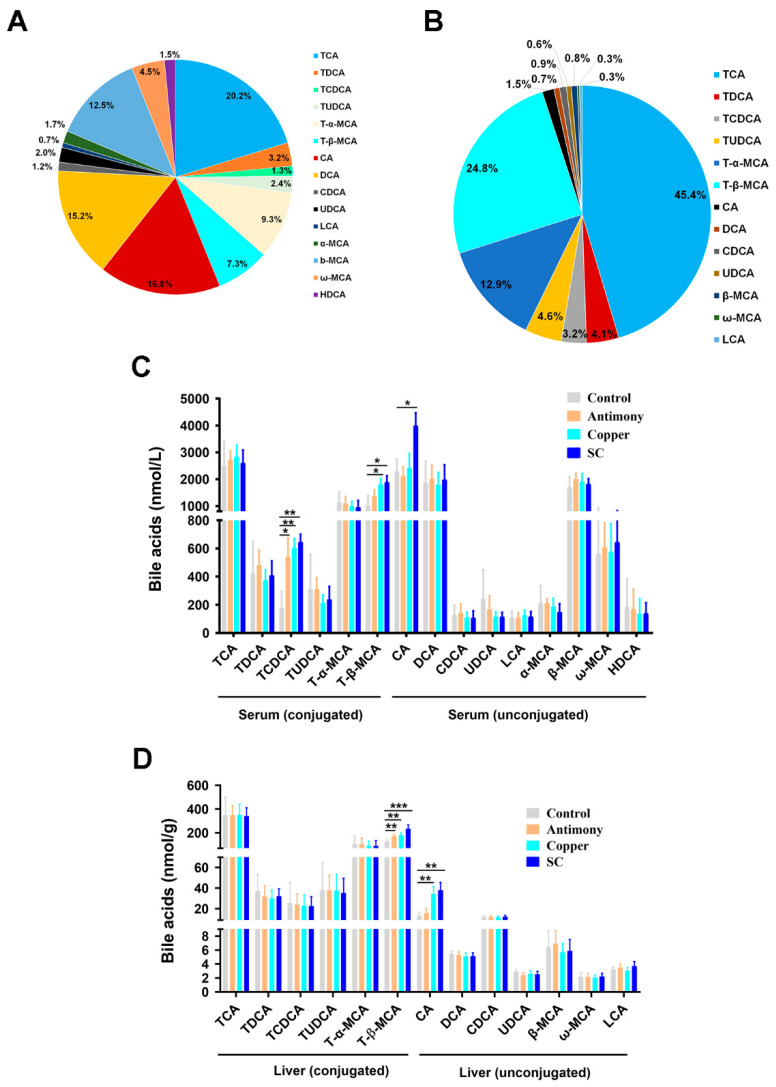
Quantification of unconjugated and conjugated BAs in serum and liver was performed using UHPLC-QQQ-MS across the control, the antimony, the copper, and the SC groups. (**A**) Percentage of serum BAs in the control group. (**B**) Percentage of hepatic BAs in the control group. (**C**) Concentrations of unconjugated and conjugated BAs in serum. (**D**) Concentrations of unconjugated and conjugated BAs in liver. Data are expressed as mean ± SD for each group with *n* = 7. * *p* < 0.05, ** *p* < 0.01, and *** *p* < 0.001 indicate statistically significant differences determined by the two-tailed Student’s *t* test.

The targeted quantification of BAs exhibited that the unconjugated BAs (CA, 16.8%; DCA, 15.2%; *β*-MCA, 12.5%) and the conjugated BAs (TCA, 20.2%) were dominant BAs in the serum BA pool of the control group (Figure 3A). The conjugated BAs (TCA, 46.1%; T-*α*-MCA, 13.1%; T-*β*-MCA, 25.2%) in the liver BA pool of the control group were dominant BAs in the liver BA pool of the control group (Figure 3B). In serum unconjugated BAs, CA was remarkably elevated in the SC group. In serum conjugated BAs, TCDCA was significantly increased in all three treatment groups, and T-*β*-MCA was remarkably increased in the copper group and the SC group (Figure 3C). In liver unconjugated BAs, CA was markedly increased in the copper and SC groups. In liver conjugated BAs, T-*β*-MCA was significantly elevated in all three treatment groups (Figure 3D).

**Figure 4:**
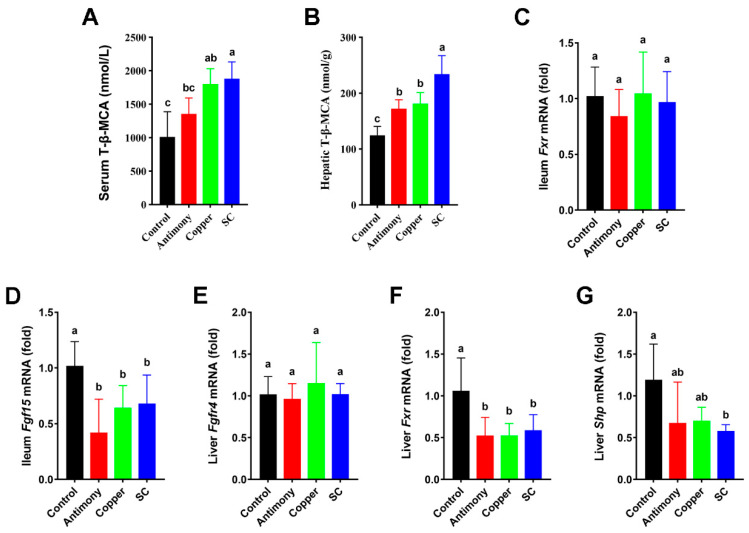
Measurements of T-*β*-MCA level and the expression levels of FXR-SHP in the liver and FXR-FGF15 in the ileum. (**A**) Serum T-*β*-MCA level. (**B**) Hepatic T-*β*-MCA level. (**C**,**D**) The mRNA expression of *Fxr* and *Fgf15* in the ileum. (**E**–**G**) The mRNA expression of *Fxr*, *Shp*, and *Fgfr4* in the liver. Data are depicted as mean ± SD for each group with *n* = 7. Bar graphs (**A**–**G**) labeled with unique letters on top denote statistically significant differences as determined by one-way ANOVA (*p* < 0.05).

Notably, serum T-*β*-MCA was significantly increased in the copper and SC groups by 1.8-fold, and hepatic T-*β*-MCA was markedly elevated in the antimony and copper groups by 1.5-fold and elevated in the SC group by 1.8-fold (Figure 4A,B). *Fxr* mRNA level in the ileum was not strikingly changed in all three treatment groups, but *Fgf15* mRNA level in the ileum was significantly reduced in the copper and SC groups by 1.7-fold and reduced in the antimony group by 2.2-fold (Figure 4C,D). The *Fgfr4* mRNA level in the liver was also not remarkably altered in all three treatment groups, whereas the hepatic *Fxr* mRNA level was significantly reduced in all three treatment groups by 2-fold, and *Shp* mRNA level was remarkably reduced in the SC group (Figure 4E–G).

### 2.3. Gut Microbiota Analysis

**Figure 5:**
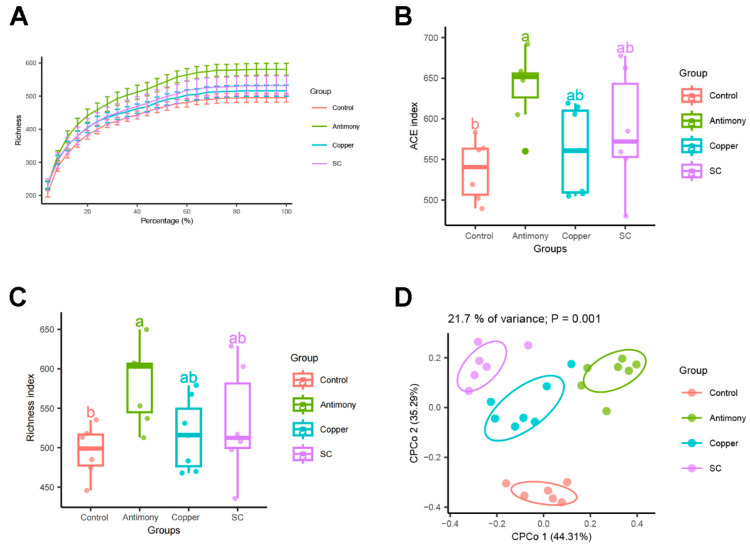
Analysis of gut microbial diversity and community structure from the control, the antimony, the copper and the SC groups. (**A**) The rarefaction curve of taxonomic units’ richness. (**B**) *α*-Diversity based on the ACE index. (**C**) *α*-Diversity based on the richness index. (**D**) *β*-diversity was visualized through CPCoA plot derived from the Bray−Curtis matrix. The data are expressed as mean ± SD for each group of *n* = 7. Bar charts (**B**,**C**) with distinct letters on top signify statistically significant outcomes determined by one-way ANOVA (*p* < 0.05).

Utilizing high-throughput sequencing, we assessed the gut microbiota composition caused by antimony and/or copper exposure. The rarefaction curve, which measures species richness, reached a plateau (Figure 5A), suggesting adequate sequencing depth. In comparison to the control group, the *α*-diversity indices such as the ACE and richness indices were markedly elevated in the antimony group (Figure 5B,C). Regarding *β*-diversity, the constrained principal coordinate analysis (CPCoA) based on the Bray–Curtis dissimilarity matrix revealed significant inter-group variations (*p* = 0.001, Figure 5D).

**Figure 6:**
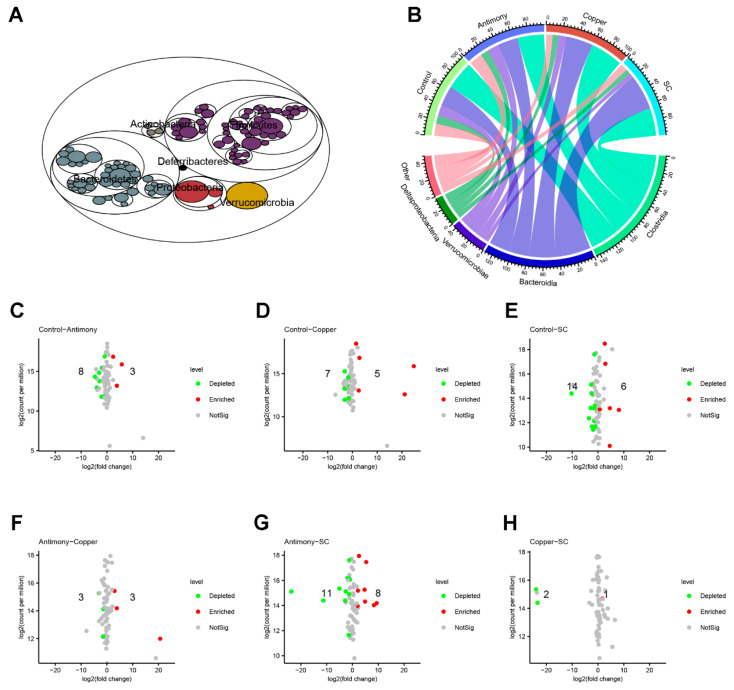
Dominant taxa at different levels of gut microbiota and the volcano plots of differential genera between pairwise comparisons. (**A**) The maptree of relative abundance of gut bacteria at the phylum level. (**B**) Circle plot at the class level. (**C**) Control group vs. Antimony group. (**D**) Control group vs. Copper group. (**E**) Control group vs. SC group. (**F**) Antimony group vs. Copper group. (**G**) Antimony group vs. SC group. (**H**) Copper group vs. SC group. Data are presented as the mean ± SD for each group of *n* = 7.

At the phylum level, the maptree diagram confirmed that the dominant bacterial groups were Bacteroidetes, Firmicutes, Verrucomicrobia, and Proteobacteria (Figure 6A). At the class level, the chord diagram indicated that the leading classes were Clostridia, Bacteroidia, Verrucomicrobiae, and Deltaproteobacteria (Figure 6B). Volcano plots identified genera with significant variations across groups (Figure 6C–H). Between the control and antimony groups, 11 genera exhibited remarkable differences, with 3 up and 8 down. The control and copper groups exhibited 12 differential genera, with 5 up and 7 down. The control and SC groups exhibited 20 different genera, with 6 up and 14 down. The antimony and copper groups had six differential genera, with three up-regulated and three down-regulated. The antimony and SC groups showed 19 different genera, with 8 up and 11 down. The copper and SC groups exhibited three different genera, with one up and two down.

### 2.4. Differential Bacterial Genera and the Relationships Between Lactobacillus and BAs

**Figure 7:**
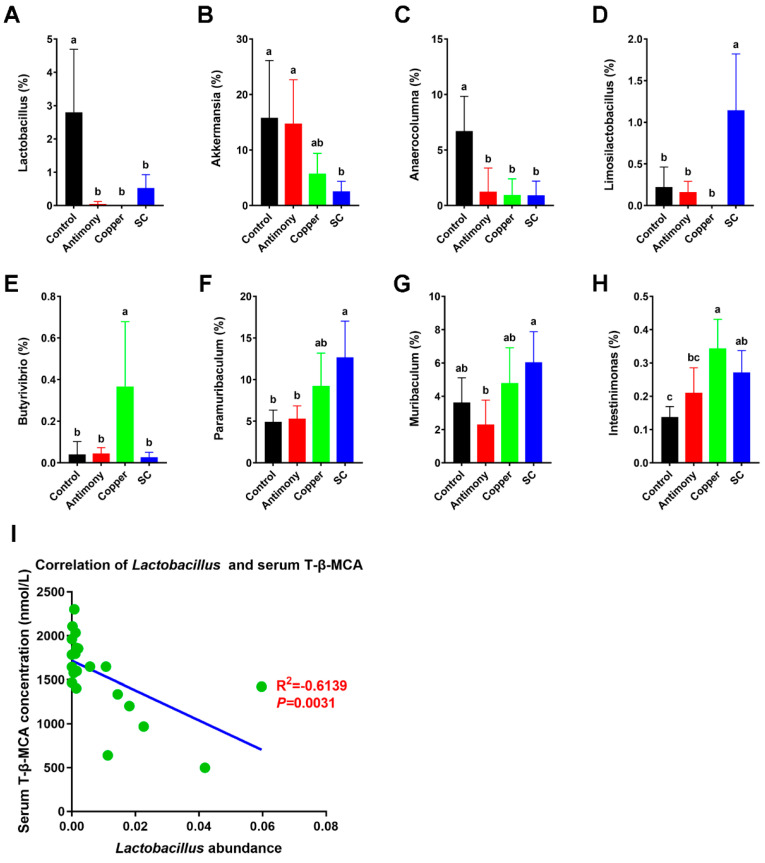
Differential bacterial genera and the relationships between *Lactobacillus* spp. and BAs. (**A**–**H**) Representational differential genera include *Lactobacillus* spp., *Akkermansia* spp., *Anaerocolumna* spp., *Limosilactobacillus* spp., *Butyrivibrio* spp., *Paramuribaculum* spp., *Muribaculum* spp., and *Intestinimonas* spp. (**I**) The correlation scatter plots between *Lactobacillus* spp. and serum T-*β*-MCA. Data are presented as the mean ± SD for each group of *n* = 7. Bar charts (**A**–**H**) with different letters on top indicate statistically significant results based on Wilcox analysis (*p* < 0.05, FDR < 0.1).

There were significant differences in the bacterial genera after antimony and/or copper treatment, with the down-regulation of beneficial bacteria (*Lactobacillus* spp., *Akkermansia* spp., and *Anaerocolumna* spp.) (Figure 7A–C), as well as the up-regulation of harmful bacteria (*Limosilactobacillus* spp., *Butyrivibrio* spp., *Paramuribaculum* spp., *Muribaculum* spp., and *Intestinimonas* spp.) (Figure 7D–H). Previous studies have shown that the down-regulation of *Lactobacillus* spp. abundance induced an up-regulation of the level of the FXR antagonist T-*β*-MCA [25]. Notably, *Lactobacillus* spp. abundance was significantly reduced in antimony- and/or copper-treated groups by 2.6-fold compared to the control group (Figure 7A). The correlation scatter plots between *Lactobacillus* spp. and serum T-*β*-MCA were conducted based on the data from the control, antimony, copper, and SC groups. The results showed that the abundance of *Lactobacillus* spp. was markedly negatively correlated with the levels of serum T-*β*-MCA (R^2^ = −0.6139, *p* = 0.0031) (Figure 7I).

## 3. Discussion

Urban agriculture is critical to ensuring long-term food security [26]. However, the increasingly severe air and soil pollution in urban areas, as well as the transfer of metal contaminated soil and pollutants to vegetables, cause health and safety risks related to urban agriculture [27]. Antimony and copper are the most common carcinogens and hepatotoxic agents [28,29]. Antimony exposure can cause lipid metabolism disorder and steatosis in liver tissue, lead to hepatic energy metabolism abnormality and oxidative stress [9], and endoplasmic reticulum stress induced by copper can promote liver apoptosis in mice [10]. In this study, the raised levels of serum ALP and UREA, as well as liver H&E staining results, revealed that antimony and/or copper could produce liver inflammation. This was further verified by the enhanced hepatic inflammatory factor IL-1*β*. Abnormal in terms of levels and the secretion of BAs can cause hepatic inflammation or liver apoptosis [13]. As we know, cholesterol in liver cells can be converted to BAs. In our study, serum CHO and hepatic CHO were down-regulated, and serum TBA was up-regulated in antimony- and/or copper-treated groups, confirming that antimony and/or copper can cause BA abnormalities. However, the mechanism of BA’s effect on liver inflammation induced by antimony and/or copper has been unclear so far. This study focused on the targeted metabolomics of BAs, high-throughput sequencing, qPCR, and ELISA to investigate the effects of antimony and/or copper on BA metabolism and gut microbiota in mice.

Diet can significantly affect the composition and function of the gut microbiota, thereby affecting overall health and disease status, including liver inflammation [30]. The Mediterranean diet is rich in fiber and polyunsaturated fatty acids (PUFAs), which can promote the growth of beneficial bacteria, such as *Lactobacilli* spp. and *Bifidobacteria* spp., while reducing pathogenic bacteria [31]. The microbiota-modifying compounds and antimicrobial phytochemicals, such as capsaicin, quercetin, and other flavonoids, can significantly affect gut microbiota and liver inflammation. Capsaicin has a relieving effect on liver inflammation by regulating the gut microbiota, reducing the number of pathogenic bacteria, and increasing the abundance of beneficial microorganisms [32,33]. Quercetin regulates gut microbiota by increasing beneficial bacteria to relieve inflammation [34]. These compounds can improve gut microbiota composition, reduce oxidative stress, and prevent liver inflammation by regulating the gut liver axis.

BAs are produced in the liver from cholesterol and then converted to secondary BAs by gut microbiota [35]. BAs regulate intestinal homeostasis and inflammation. Studies have shown that the occurrence of liver inflammation is associated with the increase in TBA concentration [36]. In our study, serum TBA was increased in antimony- and/or copper-treated groups, and hepatic TBA was increased in the SC group, and serum CHO and hepatic CHO decreased, indicating the BA biosynthesis was elevated in the liver of antimony- and/or copper-treated mice. This result could be further supported by the increased expression of hepatic *Cyp7a1* mRNA in the SC group, which is a key enzyme for converting cholesterol into BAs in the liver [37]. Most of the primary BA biosynthesis pathways are initiated by the rate-limiting enzyme *Cyp7a1* [36]. In summary, liver inflammation caused by antimony and/or copper may be attributed to the elevated TBA level and hepatic *Cyp7a1* mRNA and the decreased CHO level.

In general, gut microbiota are triggers of host inflammation [38]. Therefore, we focused on the changes in gut microbiota. Our study found no significant difference in α-diversity of bacteria between paired comparisons. However, there was a significant difference in *β*-diversity (*p* = 0.001), indicating that antimony- and/or copper-disrupted gut microbiota structure. As beneficial bacteria, the down-regulation of *Lactobacillus* spp., *Akkermansia* spp., and *Anaerocolumna* spp. are closely related with liver inflammation [39,40]. As harmful bacteria, the up-regulation of *Limosilactobacillus* spp., *Butyrivibrio* spp., *Paramuribaculum* spp., *Muribaculum* spp., and *Intestinimonas* spp. [41,42] is closely related with liver inflammation. In this study, the abundance of *Lactobacillus* spp., *Akkermansia* spp., and *Anaerocolumna* spp. was markedly reduced in the antimony and/or copper groups (Figure 7A–C), and the abundance of *Limosilactobacillus* spp., *Butyrivibrio* spp., *Paramuribaculum* spp., *Muribaculum* spp., and *Intestinimonas* spp. was significantly elevated (Figure 7D–H).

BAs are steroidal acids that play critical roles in digestion and metabolism. The enterohepatic circulation of BAs from the liver to the intestine and back to the liver plays a central role in metabolic regulation and homeostasis [13]. Abnormal BAs may cause inflammation, apoptosis, and cell death [43]. Primary BAs play a key role in cholesterol metabolism and host–microbial interactions [44]. In our study, antimony- and/or copper-treated groups up-regulated the level of CA in serum and liver. T-*β*-MCA as the primary bile acid is metabolized from *α*-MCA in the liver, and T-*β*-MCA is a risk factor for inflammation [45]. T-*β*-MCA is increased in murine models of inflammation, accompanied by the down-regulation of *Lactobacillus* spp. [25]. Similarly, our results showed that serum T-*β*-MCA and hepatic T-*β*-MCA were significantly up-regulated, and *Lactobacillus* spp. was markedly down-regulated in antimony- and/or copper-treated groups. The up-regulations of T-*β*-MCA contributed to BA abnormalities induced by antimony and/or copper.

**Figure 8:**
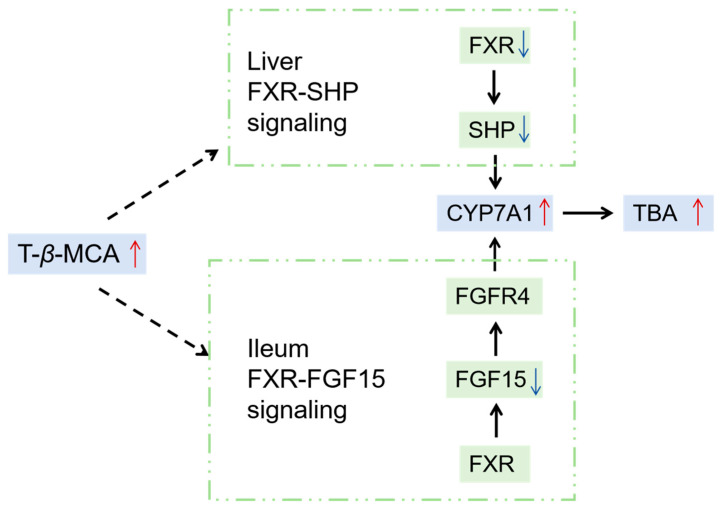
The signaling/metabolic pathways of BAs in this study.

BAs can activate FXR, a nuclear receptor found in the enterohepatic circulation. Recent research has demonstrated that the bacterial metabolism of BAs can influence FXR signaling in the gut by varying the concentration and composition of FXR agonists and antagonists. T-*β*-MCA is considered an FXR antagonist, in which the level of FXR antagonist T-*β*-MCA increases, leading to the inhibition of FXR signaling [46]. In this study, antimony and/or copper treatment up-regulated T-*β*-MCA and down-regulated hepatic *Fxr*. The above-mentioned view can also be confirmed by the down-regulation of ileal *Fgf15* and hepatic *Shp* as a linear target gene of *Fxr*. FXR is considered to be a feedback regulation mechanism of CYP7A1, the rate-limiting enzyme for BA production. The down-regulation of BA-related receptors such as FGF15, SHP, and FXR are accompanied by the promotion of CYP7A1 expression [47]. As shown in Figure 8, the signaling/metabolic pathways of BAs in this study were mainly the FXR-SHP pathway and the FXR-FGF15 pathway. The FXR-SHP pathway in the liver can inhibit CYP7A1 enzyme activity, thereby reducing the synthesis of BAs [48]. FXR can promote ileum FGF15 to flow into the liver, thereby inhibiting the enzyme activity of CYP7A1 [49]. Ileal FGF15 mainly inhibits CYP7A1 expression by binding to the FGFR4 receptor and the SHP nuclear receptor [50,51]. In this study, antimony and copper inhibited BA receptors such as FXR and SHP in liver and then promoted the transcription of rate-limiting enzyme CYP7A1. Antimony and/or copper suppressed the expression of *Fgf15* in the ileum. In our study, ileum *Fxr* and hepatic *Fgfr4* remained unchanged, ileum *Fgf15*, hepatic *Fxr*, and *Shp* were down-regulated, and *Cyp7a1* was up-regulated, ultimately leading to the up-regulation of TBA. Therefore, antimony and/or copper promoted liver inflammation in mice by regulating FXR-SHP and FXR-FGF15 signaling pathways, and the SC group was more obvious.

## 4. Materials and Methods

### 4.1. Reagents and Chemicals

Antimony solution, copper solution, and antimony copper solution were prepared by dissolving potassium antimony tartrate and copper sulfate in pure water, respectively. Dissolving potassium antimony tartrate (≥99.5%, cat no. XW2830074501) and copper sulfate (≥99.0%, cat no. 10008216) were purchased from China National Pharmaceutical Group Corporation Co., Ltd. (Beijing, China). Heparin sodium (≥99.5%, cat no. A603251) was purchased from Sangon Biotech Co., Ltd. (Shanghai, China). All BA standards, particularly isoflurane (≥98.0%, cat no. 792632), were purchased from Sigma-Aldrich Chemical Co., Ltd. (St. Louis, MO, USA). Acetonitrile (MeCN) (≥99.99%, cat no. 85188), HPLC-grade water (≥100%, cat no. TS-51140), and methanol (MeOH) (≥99.0%, cat no. A955-4) were purchased from Thermo Fisher Scientific Co., Ltd. (Waltham, MA, USA).

### 4.2. Animal Experiments

The study adhered to the ethical guidelines set by the ethics committee of the College of Pastoral Agriculture Science and Technology at Lanzhou University, with the research ethics approval being detailed in the Supporting Information. A total of twenty-eight male C57 BL/6 mice, weighed approximately 20 g and aged six weeks, were sourced from GemPharmatech Co., Ltd. in Nanjing, China. The housing conditions were maintained with a humidity level of 40–60%, a temperature of 22 ± 1 °C, and a light/dark cycle of 12 h each, under no specific-pathogen-free (SPF) conditions. In our experiment, the mice in the barrier animal facilities were first domesticated for about a week to adapt to the breeding environment. Subsequently, the mice were randomly assigned to four groups, each consisting of seven individuals: the control group receiving sterilized water, the antimony-treated group (10 mg/kg antimony) [9], the copper-treated group (100 mg/kg body weight) [10], and the SC group (10 mg/kg antimony and 100 mg/kg copper). The mice were administered a different treatment solution via gavage every two days and were provided with a standard diet for a duration of six weeks. Body weight and food consumption were recorded weekly on Monday. At the end of the six-week period, the mice were euthanized using isoflurane anesthesia, and samples of serum, liver, ileum, and cecal contents were collected and preserved at −80 °C. For intestinal sampling, the intestine was first extracted and cleaned with sterile saline before the ileum was excised for sampling, and the cecal contents were extracted from the cecum. Given the significance of the FXR-FGF15 pathway in the ileum, which is closely associated with BAs, the ileum was selected as a key area for investigation.

### 4.3. Serum Biochemical Indicators and Histopathology of the Liver

Serum samples were collected to determine various serum biochemical parameters. These parameters included levels of total bile acids (TBA), alkaline phosphatase (ALP), aspartate aminotransferase (AST), glucose (GLU), blood urea nitrogen (BUN), triglycerides (TG), creatinine (CREA), cholesterol (CHO), total bilirubin (T-BIL), high-density lipoprotein cholesterol (HDL-C), alanine transaminase (ALT), and direct bilirubin (D-BIL). Liver samples were prepared by embedding and sectioning and then stained with hematoxylin and eosin (H&E). The clinical biochemical assays of the serum and the histopathological examination of the liver tissues were performed by Wuhan Servicebio Technology Co., Ltd., located in Wuhan, China.

### 4.4. Quantitative Real-Time PCR Analysis

Ribonucleic acid (RNA) was extracted from 100 mg samples of both frozen ileum and liver tissues using an RNA extraction kit provided by Biogoethe Biotechnology Co., Ltd., based in Wuhan, China. A quantity of 1 μg of RNA was then used to generate complementary DNA (cDNA) with a reverse transcription kit also from Biogoethe Biotechnology Co., Ltd., Wuhan, China. The specific primers for quantitative real-time polymerase chain reaction (qPCR) were detailed in the Supporting Information (Table S2). The qPCR reactions were carried out using a SYBR green-based reagent, with the following thermal profile: an initial denaturation at 95 °C for 20 s, followed by 40 cycles of 95 °C for 30 s, and 60 °C for 30 s. β-actin served as the endogenous control gene. The qPCR data analysis was conducted employing the Applied Biosystems Comparative CT method (ΔΔCT).

### 4.5. Quantification of BA Metabolites

BA metabolites were isolated from liver tissues (approximately 10 mg) and serum samples (approximately 50 μL), following the extraction method outlined in the Supporting Information. The analysis of BAs metabolites was conducted using ultra-high-performance liquid chromatography coupled with a triple quadrupole mass spectrometer (UHPLC-QQQ-MS), based on a previously established protocol with some refinements. The complete list of 28 BAs standard compounds, along with their classifications, are presented in Table S1.

### 4.6. Enzyme-Linked Immunosorbent Assay (ELISA) Analysis

Concentrations of TBA in the liver, total cholesterol (CHO) in the liver, and the pro-inflammatory cytokines interleukin-1*β* (IL-1*β*), interleukin-6 (IL-6), and tumor necrosis factor-*α* (TNF-*α*) in the serum were determined using enzyme-linked immunosorbent assay (ELISA) kits supplied by Biogoethe Biotechnology Co., Ltd., Wuhan, China.

### 4.7. High-Throughput Sequencing of Gut Microbiota

Approximately 100 mg of cecal content from the mice was used for the extraction of total DNA. The preparation of the 16S rRNA gene amplicon sequencing library was carried out according to the guidelines provided by Illumina Co., Ltd., San Diego, CA, USA. The processes of assembling and filtering data for quality, classifying bacterial characteristics, and conducting statistical analysis were comprehensively described in the Supporting Information.

### 4.8. Data Analysis

The statistical analysis and graphical representation were performed using R Programming Language (version 4.1.0) and GraphPad Prism (version 8.0). Correlations between *Lactobacillus* spp. and specific bile acids were assessed with GraphPad Prism. Significance for pairwise comparisons was set at a *p*-value threshold of less than 0.05, determined by either one-way ANOVA or two-tailed Student’s *t*-test. For identifying differential genera within the gut microbiota, volcano plots were utilized for visualization, and boxplots were generated following Wilcoxon’s rank-sum test with significance levels set at *p* < 0.05 and false discovery rate (FDR) < 0.1.

## 5. Conclusions

A brief description of the mechanism of antimony- and/or copper-induced liver inflammation was conducted. Antimony and/or copper disturbed the gut microbiota balance, leading to a decrease in *Lactobacillus* spp. level and an increase in T-*β*-MCA levels in both serum and liver. Elevated T-*β*-MCA and TBA levels in serum and liver contributed to BA abnormalities. As an FXR antagonist, T-*β*-MCA inhibited the FXR-FGF15 pathway in ileum and the FXR-SHP pathway in liver, thereby promoting the transcription of CYP7A1 and up-regulating TBA concentration. The liver inflammation induced by antimony and/or copper was mediated through alterations in gut microbiota and disruptions in BA metabolism, regulated by the FXR-SHP and FXR-FGF15 pathways. Nonetheless, the intricate molecular mechanisms behind antimony- and/or copper-induced liver inflammation need further investigation.

## Data Availability

The 16S rRNA sequences of gut microbiota have been deposited in the NCBI GenBank database with the accession numbers from PRJNA1077802.

## Supplementary Materials

The following supporting information can be downloaded at: https://www.mdpi.com/article/10.3390/ijms26073332/s1.
