# Supplementary material for: Gut Microbiota and Its Metabolite Taurine-β-Muricholic Acid Contribute to Antimony- and/or Copper-Induced Liver Inflammation
Source: Int J Mol Sci. 2025 Apr 3;26(7):3332. doi: 10.3390/ijms26073332 (PMC11989503; doi:10.3390/ijms26073332)
Supplement: Supplementary file 1 [file ijms-26-03332-s001.zip › ijms-3489014-supplementary.pdf]

Supporting Information

Materials and methods

Quantification of BAs Metabolites. The full names and classifications of 28 BAs standard compounds were listed in Supporting Information (Table S1), which were purchased from Steraloids Inc. (Newport, Rhode Island, United States). The internal standards included glycocholic acid-D4, cholic acid-D4, glycochenodeoxycholic acid-D4, chenodeoxycholic acid-D4, deoxycholic acid-D4, and lithocholic acid-D4. The samples of liver mixed with internal standard (20 μL) were respectively homogenized with 980 mL methanol: water solution (2:1 v/v) containing 0.005% formic acid using the Qiagen Tissue-Lyser (Retsch GmBH, Hannover, Germany) at 20 Hz for 90 s. The extracted supernatants were collected by centrifugation and filtered using 0.22 μm Nylon 66 Syringe filters (Jin Teng Experimental Equipment Co., Ltd, Tianjin, China). Serum sample (50 μL) was uniformly mixed with 50 μL of internal standard after 50 times dilution, and then mixed with 500 μL precooled methanol. After centrifugation for 20 m (4 °C), the supernatants were collected and evaporated to dryness. Dried extracts were reconstituted in 50 μL methanol: water solution (2:1 v/v) containing 0.005% formic acid. Qualitative and quantitative analyses of BAs metabolites were performed using an ultrahigh performance liquid chromatography (Agilent 1290) coupled with a 6460 triple quadrupole mass spectrometry (UHPLC-QQQ-MS, Agilent Technologies, Inc., Santa Clara, CA, USA). The conditions of UHPLC-QQQ-MS were as follows:

Mobile phases included A (water with 0.005% HCOOH, v/v) and B (acetonitrile with 0.005% HCOOH, v/v). The elution gradient was set step wise as follows: 1. 25% B to 33% B for 2 mins; 2. 33% B to 35% B for 4 mins; 3. 35% B to 70% B for 5 mins. The flow rate was 0.6 mL/min. MS detection of bile acids were conducted in negative mode. Fragmentor and product ion for every bile acid were optimized through direct infusion of available bile acid standards to improve detective sensitivity. The precursor ions of BAs metabolites were pre-scanned through multiple reaction monitoring (MRM) of all sample mixtures and the structures were identified through MS/MS spectra. Quantification of BAs metabolites was performed using calibration curves based on MRM and the ratios of the integrated peak areas of BAs metabolites and internal standards.

Gut microbiota analysis. About 100 mg of cecal contents from mice were extracted for total DNA using the E.Z.N.A.® soil DNA Kit (Omega Bio-tek Co., Ltd, Norcross, GA, USA). Equimolar amounts of purified amplicons were pooled and paired-end sequencing (2 × 300 bp) were performed using an Illumina MinSeq platform by Shanghai Majorbio Bio-pharm Technology Co., Ltd (Shanghai, China). After demultiplexing, the resulting sequences were merged with FLASH (v1.2.11) and quality filtered with fastp (0.19.6). Then the high-quality sequences were de-noised using Deblur plugin in the Qiime2 (version 2020.2) pipeline with recommended parameters, which obtained single nucleotide resolution based on error profiles within samples. Deblur denoised sequences are usually called amplicon sequence variants

(ASVs). In this study, these denoised sequences were assigned to bacterial features, which are synonymous to ASVs. To minimize the effects of sequencing depth on alpha and beta diversity measure, the number of sequences from each sample was rarefied to 30000, which still yielded an average Good's coverage of 97.90%. The taxonomy of these features was performed using the Vsearch consensus taxonomy classifier implemented in Qiime2 and the SILVA 16S rRNA database (version 138).

Table S1. The full names and classifications for 28 bile acid standard compounds.

Abbreviation	Full Name	Groups
T-β-MCA	Tauro-beta-Muricholic acid	Conjugated
TCA	Taurocholic acid	Conjugated
THCA	Taurohyocholic acid	Conjugated
T-α-MCA	Tauro-alpha-Muricholic acid	Conjugated
TUDCA	Tauroursodeoxycholic acid	Conjugated
TDCA	Taurodeoxycholic acid	Conjugated
TLCA	Taurolithocholic acid	Conjugated
TCDCA	Taurochenodeoxycholic acid	Conjugated
GCDCA	Glycochenodeoxycholic acid	Conjugated
GLCA	Glycolithocholic acid	Conjugated
GDCA	Glycodeoxycholic acid	Conjugated
GHDCA	Glycohyodeoxycholic acid	Conjugated
CA	Cholic acid	Unconjugated
LCA	Lithocholic acid	Unconjugated
CDCA	Chenodeoxycholic acid	Unconjugated
DCA	Deoxycholic acid	Unconjugated
HCA	Hyocholic acid	Unconjugated
UDCA	Ursodeoxycholic acid	Unconjugated
HDCA	Hyodeoxycholic acid	Unconjugated
nutriCA	Nutri cholic acid	Unconjugated
ACA	Allocholic acid	Unconjugated
iso-DCA	Iso-Deoxycholic acid	Unconjugated
iso-LCA	Iso-lithocholic acid	Unconjugated
GCA	Glycocholic acid	Unconjugated
12-ketoDCA	12-keto deoxycholic acid	Unconjugated

ω-MCA	Omega-Muricholic acid	Unconjugated
α-MCA	Alpha-Muricholic acid	Unconjugated
β-MCA	Beta-Muricholic acid	Unconjugated

Table S2. Primers used for quantitative real-time PCR.

	Forward primer (5' -3')	Reverse primer (5'-3')
Fxr	TCCAGGGTTTCAGACACTGG	GCCGAACGAAGAAACATGG
Shp	CGATCCTCTTCAACCCAGATG	AGGGCTCCAAGACTTCACACA
Cyp7a1	AGCAACTAAACAACCTGCCAGT	ACTAGTCCGGATATTCAAGGATGCA
Fgf15	ACGTCCTTGATGGCAATCG	GAGGACCAAAACGAACGAAATT
Fgfr4	CTGTATGGGCTAATGAGGGAGTG	TCAGGCGGAGGTCAAGGTAC
β-actin	GTACCACCATGTACCCAGGC	AACGCAGCTCAGTAACAGTCC

Fxr: farnesol X receptor; *Shp*: small heterodimer partner; *Cyp7a1*: cholesterol 7-alpha hydroxylase; *Fgf15*: fibroblast growth factor; *Fgfr4*: fibroblast growth factor receptor.

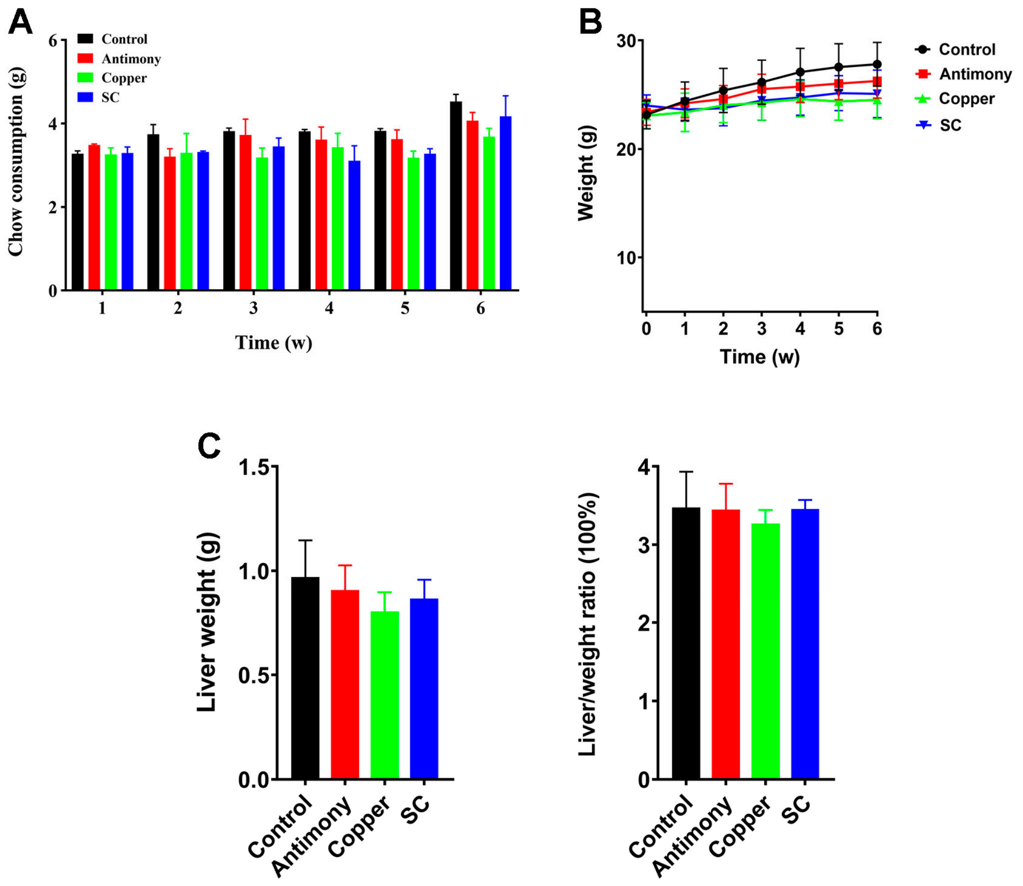

Figure S1. Effects of antimony or/and copper on chow consumption and body weight of mice. (A) Chow consumption. (B) Body weight per week. (C) Final liver weight. (D) Final liver/weight ratio.

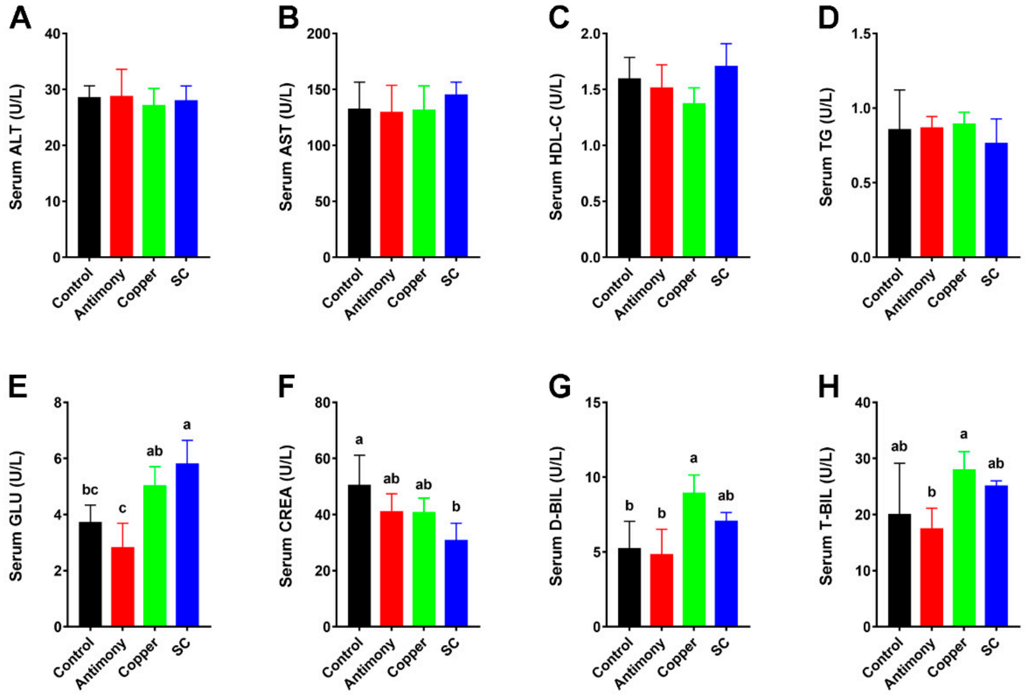

Figure S2. Serum clinical biochemical analyses in mouse liver caused by antimony or/and copper. Serum clinical biochemical analyses including serum ALT, AST, HDL-C, TG, GLU, CREA, D-BIL and T-BIL. ALT, alanine aminotransferase. AST, aspartate transaminase. HDL-C, high density lipoprotein cholesterol. TG, triglycerides. GLU, glucose. CREA, creatinine. D-BIL, direct bilirubin. TBIL, total bilirubin.

Research ethics approval

兰州大学反刍动物研究所实验动物伦理审查表

Animal Ethics Review Form of Ruminant Research Institute

(Lanzhou University)

本《动物实验方案》经过实验动物伦理委员会审核，符合动物保护、动物福利和伦理原则，符合国家实验动物福利伦理的相关规定。方案的相关信息如下：

The animal use protocol listed below has been reviewed and approved by the Animal Ethical and Welfare Committee (AEWC).

课题名称<br>Protocol Title	锑铜处理对小鼠肠道菌群和代谢的影响<br>Effects of antimony or copper on gut microbiota and metabolism in mice				
申请人<br>Applicant	武丹丹<br>Dandan Wu	职称/学位<br>Title/Degree	硕士生<br>Master	邮箱<br>E-mail	wudd2023@lzu.edu.cn
课题负责人<br>Principle Investigator (PI)	石遵计<br>Zunji Shi	职称/学位<br>Title/Degree	青年研究员<br>Researcher	邮箱<br>E-mail	shizj@lzu.edu.cn
院系（部门）<br>Department	草地农业科技学院<br>College of Pastoral Agriculture Science and Technology			申请日期<br>Application date	2023.03.01
动物种类<br>Species	C57BL/6J 小鼠<br>C57BL/6J Mus musculus			动物数量<br>Quantity	32
计划执行时间<br>Period of Protocol	2023.5.23				
审查意见<br>Results of inspection	■ 符合动物福利伦理要求，可以进行实验 Agree<br>□ 调整方案后，可以进行实验 Agree after modify				
申请日期<br>Application Date	2023.03.01				

兰州大学反刍动物研究所实验动物伦理委员会

Animal Ethical and Welfare Committee of Ruminant Research Institution (LZU)

主任 (Chairman): [illegible] 日期 (Date): 2023. 3. 1
